# Polymer Doping as a Novel Approach to Improve the Performance of Plasmonic Plastic Optical Fibers Sensors

**DOI:** 10.3390/s23125548

**Published:** 2023-06-13

**Authors:** Rosalba Pitruzzella, Riccardo Rovida, Chiara Perri, Alessandro Chiodi, Francesco Arcadio, Nunzio Cennamo, Laura Pasquardini, Lia Vanzetti, Michele Fedrizzi, Luigi Zeni, Girolamo D’Agostino

**Affiliations:** 1 Department of Engineering, University of Campania Luigi Vanvitelli, 81031 Aversa, Italy; rosalba.pitruzzella@unicampania.it (R.P.); riccardo.rovida@unicampania.it (R.R.); francesco.arcadio@unicampania.it (F.A.); nunzio.cennamo@unicampania.it (N.C.); luigi.zeni@unicampania.it (L.Z.); 2 Moresense SRL, Filarete Foundation, Viale Ortles 22/4, 20139 Milano, Italy; c.perri@moresense.tech (C.P.); a.chiodi@moresense.tech (A.C.); 3 Indivenire SRL, Via Alla Cascata 56/C, 38123 Trento, Italy; l.pasquardini@indiveni.re; 4 Bruno Kessler Foundation, Center for Sensors and Devices, Via Sommarive 18, 38123 Trento, Italy; vanzetti@fbk.eu (L.V.); mfed@fbk.eu (M.F.)

**Keywords:** polymer doping, plastic optical fiber (POF), surface plasmon resonance (SPR), molecularly imprinted polymers (MIPs), iron oxide

## Abstract

In this work, $Fe_{2}O_{3}$ was investigated as a doping agent for poly(methyl methacrylate) (PMMA) in order to enhance the plasmonic effect in sensors based on D-shaped plastic optical fibers (POFs). The doping procedure consists of immerging a premanufactured POF sensor chip in an iron (III) solution, avoiding repolymerization and its related disadvantages. After treatment, a sputtering process was used to deposit a gold nanofilm on the doped PMMA in order to obtain the surface plasmon resonance (SPR). More specifically, the doping procedure increases the refractive index of the POF’s PMMA in contact with the gold nanofilm, improving the SPR phenomena. The doping of the PMMA was characterized by different analyses in order to determine the effectiveness of the doping procedure. Moreover, experimental results obtained by exploiting different water–glycerin solutions have been used to test the different SPR responses. The achieved bulk sensitivities confirmed the improvement of the plasmonic phenomenon with respect to a similar sensor configuration based on a not-doped PMMA SPR-POF chip. Finally, doped and non-doped SPR-POF platforms were functionalized with a molecularly imprinted polymer (MIP), specific for the bovine serum albumin (BSA) detection, to obtain dose-response curves. These experimental results confirmed an increase in binding sensitivity for the doped PMMA sensor. Therefore, a lower limit of detection (LOD), equal to 0.04 μM, has been obtained in the case of the doped PMMA sensor when compared to the one calculated for the not-doped sensor configuration equal to about 0.09 μM.

## 1. Introduction

Surface Plasmon Resonance (SPR) is an efficient optical sensing technique used to evaluate the refractive index (RI) variation of a dielectric medium in contact with a metal nanofilm. This phenomenon is caused by the interaction between incident light and the free electrons of a noble metal film (e.g., gold) at the metal–dielectric interface [1,2].

Typically, one of the most adopted experimental configurations to trigger the SPR phenomenon is based on prism coupling (such as in the case of Kretschmann and Otto configurations) [3,4,5]. On the other hand, optical fiber-based SPR sensors denoted several advantages over the conventional prism-based SPR sensor, such as miniaturized dimensions, low-cost, great portability, and the possibility of remote and in situ sensing [6,7,8,9,10]. SPR has been exploited in recent years, along with both glass and plastic optic fibers (POFs), to create sensitive and low-cost sensors used in several different fields, especially in biochemical sensing, and in most cases coupled with either a biological or a synthetic receptor chosen for the specific analyte of interest [6,7,11,12,13]. The interaction between the receptor and the analyte changes the refractive index of the outer layer, a modification that can be monitored, for instance, by SPR transducers. The thickness of the receptor layer, the kind of receptor (e.g., nanoMIPs, antibodies, MIP films, etc.), and the nature and size of analyte influence how much the refractive index changes when the binding occurs [1,2].

Along this line, the optical fiber structure can be opportunely modified to trigger the SPR phenomenon, such as in the case of D-shaped fibers, tapered fibers, U-bent fibers, hetero-core structured fibers, and so on [14,15,16,17,18].

One drawback of these sensors is that the sensitivity decreases exponentially with the distance from the metal–dielectric interface, as the effective interaction length is usually no larger than a few hundred nanometers [1]. A possible solution is the enhancement of the electromagnetic field, thus improving the overall performance. To this purpose, several strategies have been used in recent years to enhance the capabilities of SPR sensors based on optical fibers, with remarkable results. In this respect, Cennamo et al. [8] presented an SPR platform based on POFs, which includes an intermediate layer (a photoresist buffer layer), with a refractive index higher than that of the POF core (PMMA), between the D-shaped area and the upper gold nanofilm. Different kinds of photoresists were also investigated to determine the one who achieved the best improvement in terms of both sensitivity and signal-to-noise ratio [9]. However, this approach presents many disadvantages, first and foremost, the depositing method, based on spin-coating. In fact, this technique is time-consuming, and only one sensor at a time can be spin-coated; furthermore, the layer thickness can be too high, thus hindering the sensitivity of the sensor and, finally, different states of photoresist ageing can also influence the sensor performance [10].

In general, performance improvement can be emphasized by reducing the intermediate layer’s thickness and increasing its refractive index. To this aim, a possible alternative is represented by ultrathin graphene layers or metal oxides (MOs) films which have been commonly used as overlayers (upon the gold surface) to achieve better plasmonic performance [19,20,21,22,23]. However, in these cases, the technique has critical issues, which typically require accurate control of the thicknesses and do not always guarantee good adhesion with the substrate. Furthermore, the possibility of cross-contamination that can occur during the deposition phase using different targets should not be underestimated either.

Recently, Cennamo et al. [24] investigated MOs materials as intermediate layers (instead of photoresist buffer layers) to enhance the plasmonic phenomenon, particularly by depositing ultrathin MOs bilayers between the exposed POF core (PMMA) and the upper gold nanofilm. Nevertheless, this option still relied on spin-coating as a deposition method with many disadvantages [24]. Other approaches to improve SPR-based sensor performance can be exploited, such as employing nanoassemblies of different shapes and materials to enhance the plasmonic resonance (as described in Bhaskar et al.’s work [25]) and the use of photonic crystals coupled with fluorescence-based techniques [26]. Even though both methods have been widely used, there are many disadvantages to consider, such as the need for extremely low temperatures for nanoassemblies synthesis, while labelling of analytes with fluorophores and other photon-emitting probes can lead to poor reaction yields or long reaction times. Cunha et al. [27] investigated the doping of silica optical fiber by inserting germanium dioxide defects in the silica core, obtaining an increase in sensitivity and an extension of the range of possible applications. However, one limitation of this approach is the cost of germanium dioxide.

Another possible solution is polymer doping. Polymers, especially thermoplastics such as PMMA or PVC, have become an important alternative to metals in engineering applications due to low weight, high specific strength, chemical resistance, cheap and efficient manufacturing processes, and their physical properties. For example, a polymer-based composite was investigated by Elsheik [28] for energy harvesting; also, manufacturing has become a very important sector for polymers applications [29]. Polymeric materials have become indispensable in many industries, therefore understanding how the imposed workload impacts them (such as crack propagation [30]) and how to avoid these problems is crucial. Polymer doping has been thoroughly investigated and established to improve many properties, from mechanical to optical ones [31]. It can be performed with either organic substances, as done by Deshmukh et al. [32], or with inorganic ones, as investigated by Gavade et al. [33], Horinouchi et al. [34], and by Zhang et al. [35], or both; polymers blend doping has also been investigated by Soman et al. [36] and by Velasco et al. [37]. Among inorganic substances, Transition Metals Halides (TMH), especially chlorides, have attracted a lot of attention because of their ability to remarkably improve polymer properties [38,39], such as optical ones, according to Tawansi et al. [40]. Metal oxide nanoparticles have also been studied as doping agents [41]. However, many investigations of doped polymers have been performed on thin films, meaning that both polymer and doping agents must be dissolved in the same solvent and later repolymerized. This strategy does not apply to large-scale production, such as on an industrial level.

In this study, the authors aimed to develop a different approach to polymer doping with metal halides. Polymer doping procedures previously reported [32,33,34,35,36,37,38,39,40] were carried out by dissolving both polymer and doping agents in the same solvent, and then repolymerization as thin films was achieved. This approach does not suit our needs as PMMA acts as a waveguide, and repolymerization could alter its properties, thus hindering the sensor’s functioning and applicability. The authors have developed a doping technique applied to an already completed optical fiber and carried out by immersing the POF platform in a solution.

Premanufactured plastic holders with a D-shaped POF already embedded into them were partially immersed in a ${Fe}^{3+}$ hydroalcoholic solution for different periods to establish the optimal doping condition and let the PMMA surface adsorb the iron. The doping solution is $O_{2}$ saturated during the procedure to guarantee the formation of iron oxide soon after the adsorption.

Iron (III) oxide, whose refractive index is high, was chosen as a doping agent because it can be derived from cheap and readily available iron chloride, which is soluble in ethanol. Furthermore, $Fe_{2}O_{3}$ is readily absorbed by PMMA. So, the procedure described allows for cheap and easy industrial implementation.

Surface characterization was performed by Scanning Electronic Microscope (SEM), elemental analysis, and X-ray Photoelectron Spectroscopy (XPS) to investigate the treatment’s effect on the polymeric material.

Then, after a thin gold film was sputtered on the treated platforms (at different doping times), optical characterizations were carried out to assess the change in bulk sensitivity with respect to a not-treated SPR platform. Finally, doped and not-treated SPR platforms were functionalized with a molecularly imprinted polymer (MIP), specific for bovine serum albumin (BSA) detection, to investigate if lower analyte concentrations could be determined by exploiting the sensor based on PMMA doping procedure.

## 2. Materials and Methods

### 2.1. Chemicals and Reagents

${FeCl}_{3}$ was purchased from Sigma-Aldrich, Acrylamide (Aam) (CAS 79-06-1), N-tert-butylacrylamide (TBAm) (CAS 107-58-4), N,N’-methylenebisacrylamide (BIS) (CAS 110-26-9), 2-hydroxyethyl methacrylate (HEMA) (CAS 868-77-9), N,N,N’,N’-tetramethylethylenediamine (TEMED) (CAS 110-18-9), ammonium persulfate (APS) (CAS 7727-54-0), sodium dodecyl sulfate (SDS) (CAS 151- 21-3), and phosphate buffer solution 15 mM prepared from 1 M stock solution obtained from Sigma-Aldrich (Darmstadt, Germany).

Milli-Q water ((Milli-Q®, Merck KGaA, Darmstadt, Germany) was used as washing solvent and to prepare phosphate buffer solution 15 mM from the more concentrated stock solution. The human serum albumin (BSA) (CAS 9048-46-8), trypsin (CAS 9002-07-7), and ethanol 96% (CAS 64-17-5) were from Sigma-Aldrich.

Glycerin used to prepare solutions for sensors’ optical characterization was obtained from Sigma-Aldrich (Darmstadt, Germany).

### 2.2. SPR Experimental Setup

The experimental setup is based on a white light source, a spectrometer in the visible range, and an SPR-POF sensor chip. More in detail, SPR spectra were recorded with an SPR instrument (model Spectra 340_pro), SPR-POF platforms (mod RA1009), and custom software for data processing (Capture Spectrum Data Ver.2.4.7.0) developed by Moresense Srl (a Spin-off of University of Campania Luigi Vanvitelli).

### 2.3. MIP Synthesis and Platform Functionalization

The used MIP synthesis strategy is extensively described in [41]. To summarize, prior to the MIP functionalization, the doped and not-treated (acting as a control) platforms were treated with a 10% allyl thiol in 80% *v*/*v* ethanol solution and 10% *v*/*v* water. The solution was deposited on the platform for 12 h to create a monolayer on the gold surface, then each platform was flushed with MilliQ water to eliminate the thiol excess.

Aam, TBam, BIS, and HEMA were put in a vial and dissolved with the minimum volume of phosphate buffer solution under sonication, then degassed under a gentle flow of $N_{2}$ for several minutes. BSA was then added to the prepolymeric mixture, along with TEMED and APS. The mixture was dropped on the pre-functionalized platform and the polymeric layer was allowed to grow for 10 min at 25 °C.

After that, the excess of prepolymeric mixture was removed with Milli-Q water and the template was extracted by treating the platform using 100 µL of trypsin solution ${4.2\cdot 10}^{-8}$ M for 2 h at 30 °C and flushing times with 1 mL SDS 5% *w/w* solutions. Finally, the polymeric film was washed with Milli-Q water and dried. The polymeric gel that was formed had a dry residue of 10%.

### 2.4. SPR-Based Experimental Procedures

#### 2.4.1. Optical Characterization

A preliminary optical characterization of the bare SPR sensors (without receptor) was performed to test the bulk sensitivity. In particular, water–glycerin solutions with increasing RI were prepared and tested, in the range of 1.341 to 1.392. These RI values were checked by a commercial Abbe refractometer (model RMI, produced by Exacta Optech, München, Germany) before use.

#### 2.4.2. BSA Dose-Response Curve

BSA solutions with concentrations ranging from 0.05 μM to 10 μM were prepared in phosphate buffer and tested. First, 100 µL of each solution was deposited on the sensor surface and incubated for 10 min at room temperature to let the interaction between the analyte and the receptor occur.

After each incubation, the sensor’s surface was washed with Milli-Q water, and the SPR spectrum was recorded by considering phosphate buffer as a bulk solution. By using this measurement protocol, shifts caused by bulk changes or non-specific interactions were eliminated, and only the shift of the resonance wavelength caused by the specific analyte–receptor binding was observed.

### 2.5. Surface Characterization

The chemical characterization was performed using X-ray Photoelectron Spectroscopy (XPS). A Kratos AXIS UltraDLD instrument (Kratos, Manchester, UK) equipped with a monochromatic Al Kα (1486.6 eV) X-ray source was used to analyze the untreated fiber and the treated one in two different areas, one visually flat and one showing superficial aggregates. For the take-off angle, the angle between the analyzer’s axis and the normal to the sample surface, we used 0°, 45°, and 60°, sampling a depth ranging between 10 nm to 5 nm. For both samples, the survey spectra showing the chemical species present on the samples have been acquired. Subsequently, the core lines of the main elements present were collected with higher energy resolution. The relative elemental percentage was obtained by integrating the area under the core lines, applying the Shirley background subtraction, and correcting for the atomic sensitivity factors through a dedicated software [42]. Spectra were aligned to the position of C-C/C-H at 285 eV.

PMMA-modified fibers were morphologically analyzed by a Scanning Electron Microscopy (SEM) after being coated with a thin layer of gold in order to compensate for the surface charging. Images were acquired using the electron column of a ThermoFisher Helios 5 CXe PFIB DualBeam, and Energy Dispersive X-Ray Analyzer (EDX) (Thermofisher, Waltham, MA, USA) was used to acquire the signal of the compositional elements from the imaged area. C, O, and Fe were the chemical species acquired.

## 3. A Novel SPR-POF Platform Based on a PMMA Doping Procedure

Despite several SPR prism-based configurations reported in the literature [3,4,5], the adopted plasmonic configuration exploits an optical waveguide (i.e., a modified POF) to match the plasmonic resonance conditions at the metal–dielectric interface.

The production process of a D-shaped SPR platform can be summarized as follows. Shortly, a POF with a 1 mm diameter (consisting of a 980 µm PMMA core and a 10 µm fluorinated cladding) was blocked into a resin support, then polished first with a 5 µm polishing paper and subsequently with a 1 µm one to take the cladding and a section of the core off.

Afterwards, 250 mL of ${FeCl}_{3}$ 38% *w*/*w* solution was prepared in a hydroalcoholic solution (10% *v/v* of ethanol and 90% Milli-Q water). Several D-shaped SPR platforms were lodged in a specifically designed holder, put in the prepared solution, and left under magnetic stirring for different periods (1 h, 3 h, and 12 h) at a controlled temperature of 25 °C. After the intended time had passed, each platform was thoroughly washed and rinsed with Milli-Q water, and then dried by nitrogen.

Finally, about 60 nm of gold was sputtered on the platforms using a CCU-010 HV High vacuum coating unit (Safematic Gmbh, Zizers, Switzerland), by applying a current of 60 mA, at 0.05 mbar of pressure, for 68 s. For the not-treated SPR-POF platforms, the gold sputtering was carried out after the polishing process.

**Figure 1:**
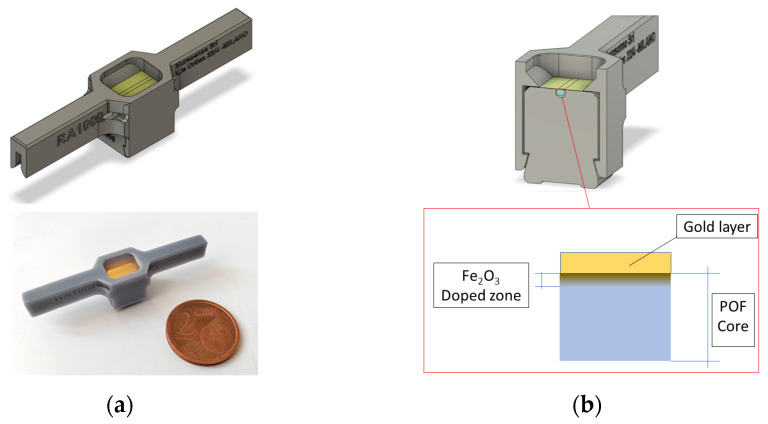
(**a**) Outline and picture of the SPR-POF platform used for the experiments. (**b**) Scheme of a cross-sectional view with a zoom of the doped SPR-POF platform after treatment.

In Figure 1, an actual image of the adopted SPR platform is reported together with a schematic cross-section highlighting the PMMA sensing area interested by the doping process.

Since the doping procedure is not performed in a nitrogen atmosphere, iron chloride can react with oxygen dissolved in the solution to create iron oxide, according to the following equation:

(1) $$4\;\mathrm{FeC}l_{3}+3O_{2}\left(g\right)\to 6\;Cl_{2}\left(g\right)↑+2\;Fe_{2}O_{3}$$

Iron oxide remains trapped in the polymer, assisted by the swelling effect induced by ethanol, and acts as a medium to increase the refractive index of the PMMA in the POF, strengthening the plasmonic phenomenon.

## 4. Experimental Results

### 4.1. Surface Characterization of the Doped PMMA

**Figure 2:**
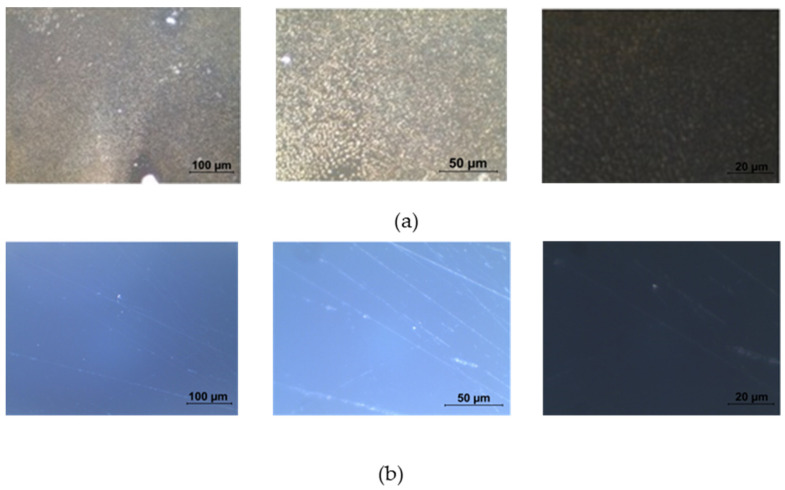
Optical microscope images of (**a**) a doped platform and (**b**) a not-treated platform in dark field taken at different magnifications (from left to right: 20×, 50×, and 100×).

Once dried, doped platforms were examined with an Axiotron microscope and reported in the following picture. The pictures in Figure 2 were acquired before gold deposition, showing remarkable differences between the doped platform (Figure 2a) and the not-treated ones (Figure 2b).

**Figure 3:**
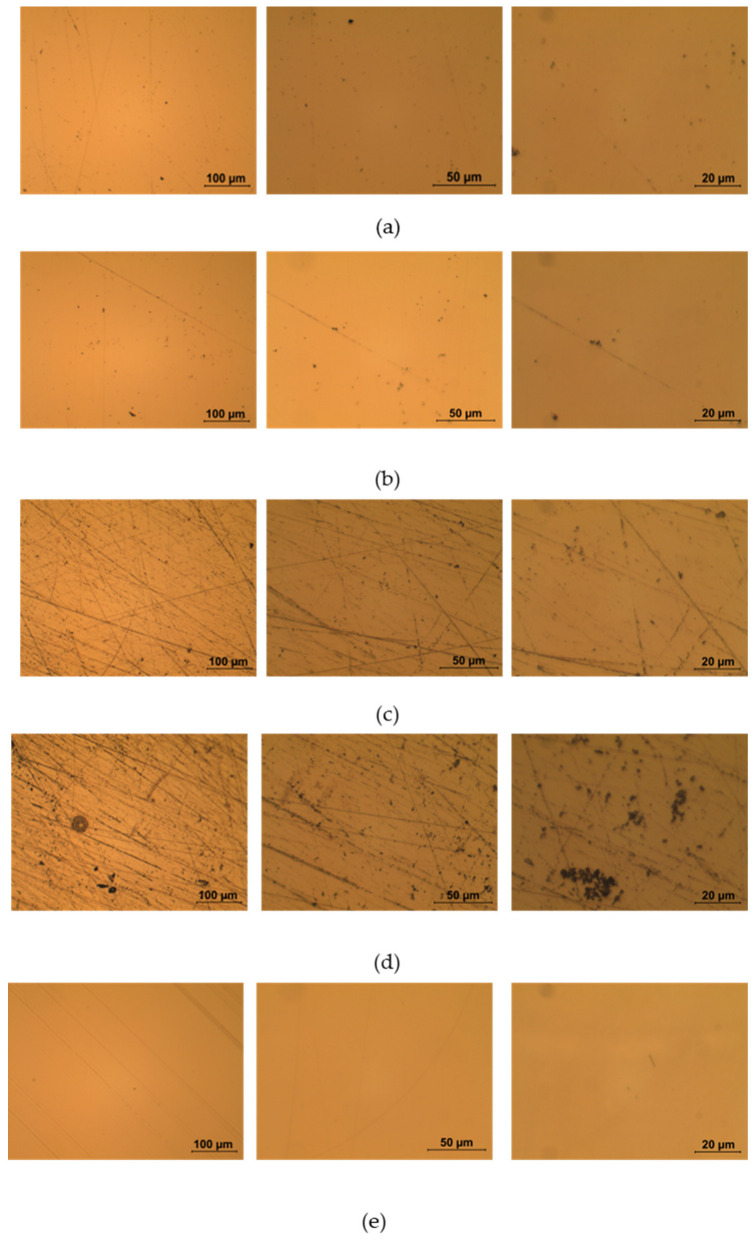
Optical microscope images with different magnification (from left to right: 20×, 50×, and 100×) of (**a**) not-treated platform, (**b**) doped platform for 1 h, (**c**) doped platform for 3 h, (**d**) doped platform for 12 h, and (**e**) photoresist-coated platform.

Figure 3 shows the surface of the different platforms after the gold sputtering process: from (a) to (d), a difference between the not-treated and the doped platforms is notable, and surface changes are more highlighted with the increasing doping time. The final image (e) displays the photoresist-coated platform realized as described in [8]. The sputtering process was not hindered by different treatment procedures. The size of the scratches (which possibly originate during the lapping procedure, as they can be seen on all the platforms regardless of doping) on the platform surface increases with the time of exposure to the solution. A possible explanation resides in a swelling of PMMA caused by ethanol in an aqueous solution [43,44,45], that magnifies the already present scratches. PMMA swelling is a useful phenomenon in this case because it helps with iron (III) absorption by the polymer matrix.

**Figure 4:**
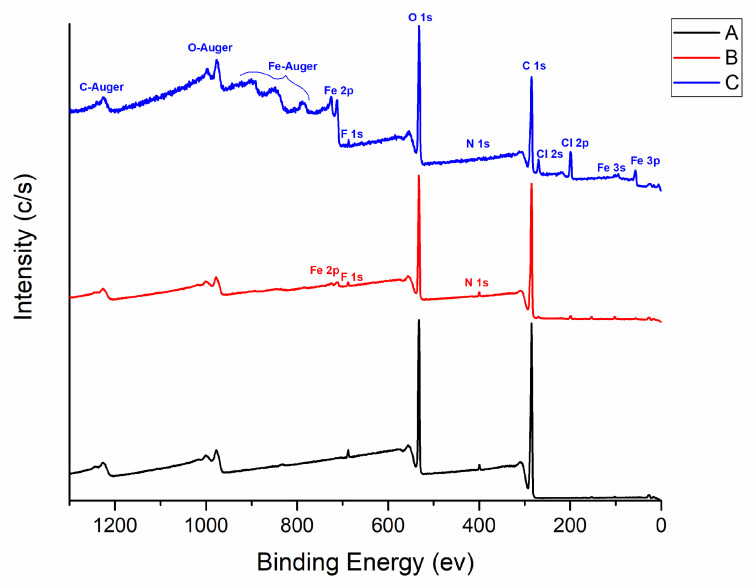
Surveys from XPS analysis on non-treated sample (A), treated without aggregates (B), or with aggregates (C). Chemical species are identified and labelled on the spectra.

After the preliminary optical microscope analysis, the chemical changes due to the doping process were monitored by means of XPS and SEM analysis. Figure 4 reports the survey spectra acquired on the PMMA fiber without any chemical treatment (A) and the main polymer components, carbon and oxygen, can be identified, with a small amount of fluorine and nitrogen (probably coming from the cladding as consequence of the lapping procedure). After the chemical doping, clear signals of iron and chlorine appeared, more evident on the sample’s areas where aggregates are present (C), but signals are also detectable on the flatter part of the sample (B).

A detailed analysis of the composition and of the oxygen core line was performed. Table 1 reports the chemical composition at three different detection angles, sampling a depth from 10 (0°) to 5 nm (60°) in order to study the penetration of the chemical treatment. As observed in Table 1, going to a more superficial analysis (60°), a clear increase in the iron content was observed both on the fiber without aggregates (B) and on the fiber with aggregates (C).

Apart from the presence of iron and chlorine on the treated fibers, a modification of the oxygen core line was observed: the two oxygen components related to the PMMA material (O2 for the O=C bonds and O3 for the C-O-C bonds) of the sample A are in good agreement with the literature [46,47]. The chemical treatment of the fibers causes the appearance of a third component at low binding energy (O1) that is related to the bonding of oxygen to iron, as also suggested by the literature [48,49]. This component is only present on the treated fibers and increases going to a more superficial analysis (60°).

The XPS analysis therefore suggests that the chemical treatment remains confined to the first nanometers of the materials.

Iron is bound only to oxygen atoms but does not insert itself in any part of the carbon chain (as demonstrated by the lack of Fe-C in XPS signals).

**Figure 5:**
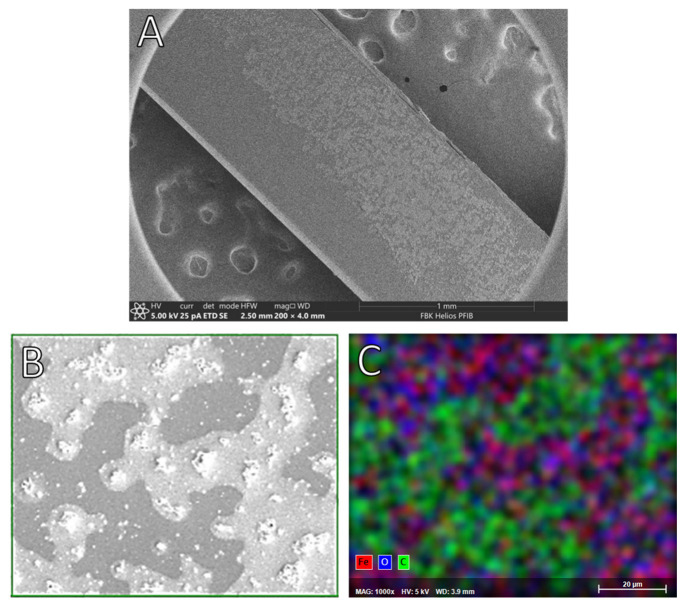
SEM image of treated fiber: (**A**) where there are clearly visible areas with aggregates. Small area analyzed with EDX technique; (**B**) and relative results; (**C**) with the overlapping of iron, oxygen, and carbon.

The presence of these aggregates was confirmed by SEM image (Figure 5A).

Figure 5B,C reports the EDX analysis on a small area of the sample; the analysis confirmed that the aggregates were mainly composed of iron and oxygen, while carbon (attributable to the PMMA core) was mainly present in the surrounding area.

### 4.2. Optical Characterization of the Doped SPR-POF Probes

A preliminary optical characterization was performed by depositing 100 µL of water–glycerin solutions on the sensor surface, with an RI ranging from 1.341 to 1.392, and recording the SPR spectra. Before each subsequent solution, the SPR surface was conditioned with the next solution to remove any leftover drops of the previous solutions.

**Figure 6:**
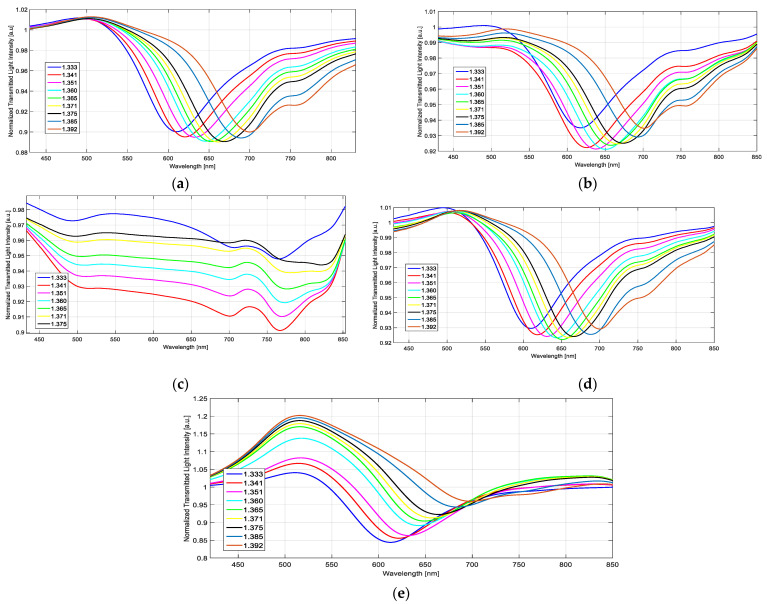
SPR spectra obtained at various external refractive indices with regards to (**a**) an SPR-POF platform doped for 1 h, (**b**) an SPR-POF platform doped for 3 h, (**c**) an SPR-POF platform doped for 12 h, (**d**) an SPR-POF non-treated platform, and (**e**) an SPR-POF platform based on non-treated PMMA covered by a photoresist layer and a gold nanofilm.

Figure 6 shows the recorded spectra concerning the not-treated SPR-POF platform (control platform), the photoresist-coated one (non-treated PMMA covered by a photoresist layer and a gold nanofilm) [8], and the treated SPR platforms (at different doping times). It should be stressed that the SPR spectra typically depend on the type of metal nanofilm, the refractive index of and the kind of light-coupling device (prism or optical fibers), and the refractive index of the external surrounding medium [8]. In the presented cases, the employed light source is a broadband one, and the SPR dip appears in the visible spectrum because of the adopted waveguide, which is a multimodal POF having a low-loss transmission in the visible region. From Figure 6, we can see how by changing the POF-based waveguide, the SPR phenomena change (shape, resonance wavelength, bulk sensitivity, etc.). More specifically, the SPR platform treated for 12 h denotes no resonance peaks (see Figure 6c), probably due to fiber damages which compromised its optical properties. For this reason, the treatment of 12 h was discarded. In fact, the interaction between the polymer matrix and ethanol in an aqueous solution leads to, as said before, swelling, and can also cause the dissolution of the polymer [43,50,51,52,53]. Moreover, iron chloride is a strong Lewis acid, so the pH of the solution may have played a role in damaging the optical fiber.

**Figure 7:**
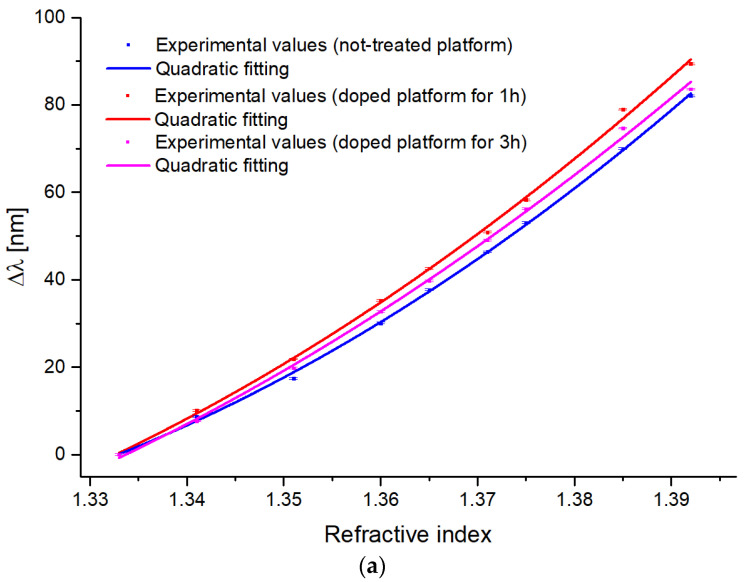

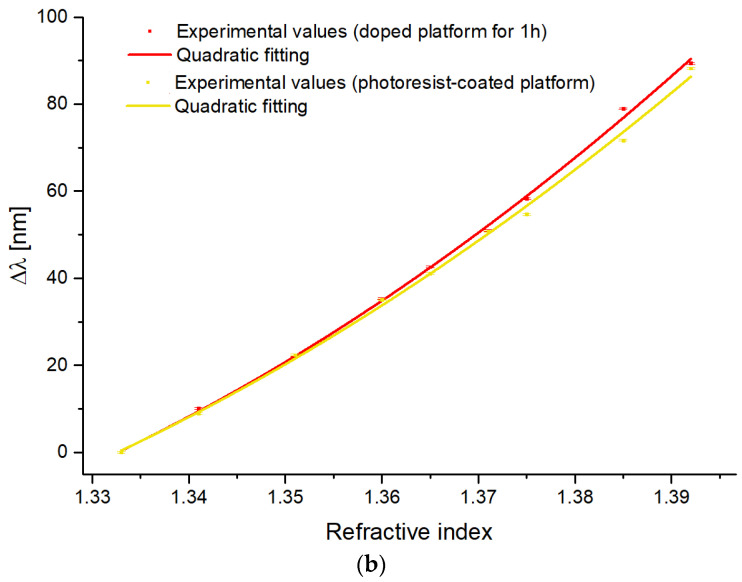
Experimental resonance wavelength variation (Δλ), calculated with respect to water (n = 1.333), versus the refractive index of the solution (error bars, SD = 0.2 nm). Quadratic fittings (solid line) are also reported for (**a**) doped and not-treated sensors; (**b**) shows a comparison between the doped sensor (1 h) with a photoresist-coated sensor configuration.

Figure 7 reports, for all the above-mentioned SPR-POF sensor configurations, the resonance wavelength variations (Δλ), calculated with respect to the water (RI = 1.333), as a function of the external refractive index (*n*). The experimental values were fitted by the quadratic function reported in Equation (2). In particular, Figure 7a shows different doped sensor chips compared with a not-treated sensor, whereas Figure 7b shows the optimized doped sensor together with a photoresist-coated sensor configuration.

Δλ = A*n*^2^ + B*n* + C (2)

where the numeric coefficients A, B, and C are reported in Table 2 for each tested sensor configuration reported in Figure 7.

In SPR sensors with a spectral interrogation, the resonance wavelength λ_res_ is determined in relation to the refractive index of the sensing layer (n). If the refractive index of the sensing layer is changed by an amount ∂n, then λ_res_ also changes by a ∂λ_res_ amount. Sensitivity (S) can be described as the shift in resonance wavelength per unit change in refractive index (nm/RIU), as indicated by the following equation:

(3) $$S\;=\frac{\partial \lambda_{\mathrm{res}}}{\partial n}\left[\frac{\mathrm{nm}}{\mathrm{RIU}}\right]$$

The sensitivity definition refers to the one adopted for SPR sensors with spectral interrogation [54,55]. So, by considering Equation (3) and the fitting equations described in Equation (2) (with the correlated parameters listed in Table 2), the bulk sensitivities can be approximated with the first derivative of the quadratic fitting functions.

**Figure 8:**
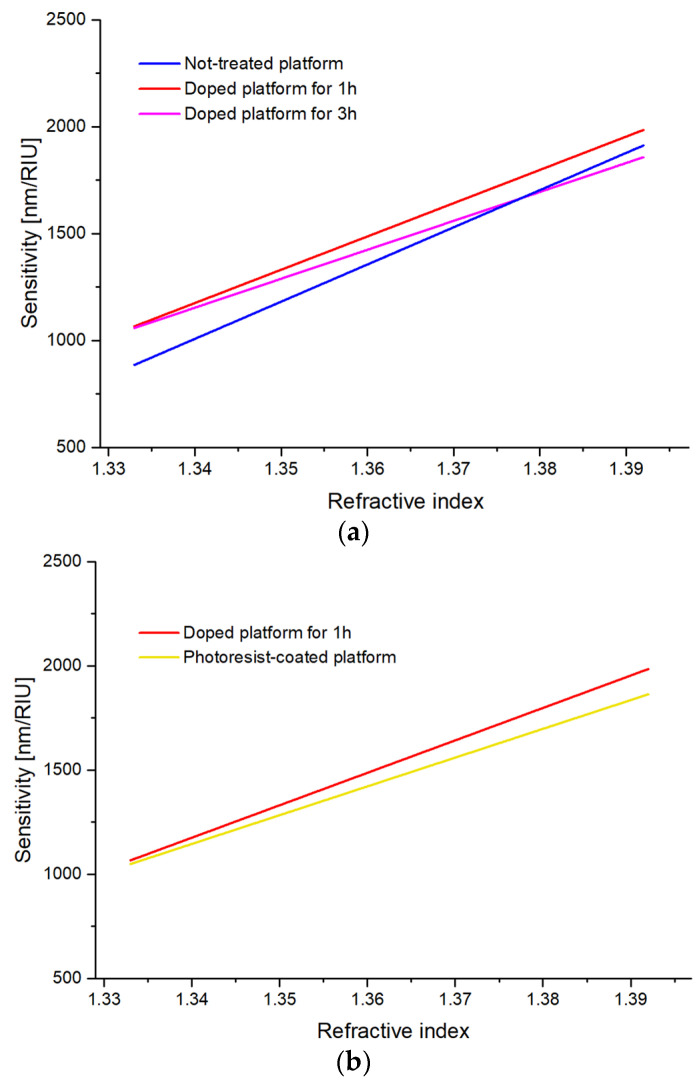
(**a**) Bulk sensitivity as a function of the refractive index for not-treated and treated (at different times) SPR sensors. (**b**) Comparison in sensitivity between the optimized doped sensor and a photoresist-coated one.

As shown in Figure 8a, the sensors treated for an hour have shown an overall higher bulk sensitivity than the other platforms, performing better in the refractive index range between 1.341 and 1.392. For comparison, Figure 8b also reports the calculated sensitivities for the photoresist-coated sensor and the one doped for 1 h. As it is clear, the bulk sensitivity of the photoresist-based sensor is lower than the optimized-treated sensor, proving that the optimized treatment with iron chloride strengthens the plasmonic phenomenon even better than the effect achieved by the photoresist-coated ones.

### 4.3. Binding Tests Exploiting Different SPR-POF Probes Combined with MIP

Overall, SPR platforms that were treated for an hour yielded the best results in terms of bulk sensitivity and were chosen to determine if lower concentrations of analytes could be detected. To this purpose, as a proof of concept, binding tests were performed by depositing upon the SPR surface of both not-treated and optimized-treated (for 1 h) platforms, an MIP layer specific for BSA detection.

**Figure 9:**
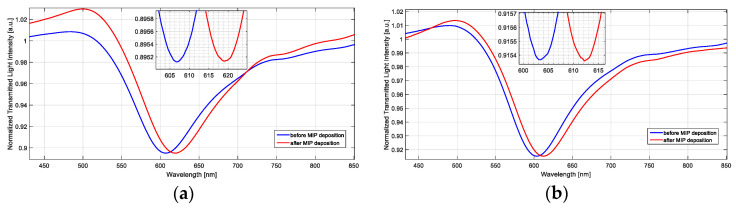
SPR spectra collected from (**a**) an optimized doped platform and (**b**) a not-treated platform, before and after the MIP deposition step.

In Figure 9, SPR spectra recorded on the same SPR platform, before and after functionalization with the MIP receptor, are shown, respectively, with a doped sensor (Figure 9a) and a not-treated one (Figure 9b). As shown in Figure 9, when the MIP layer is present on the gold surface with the same bulk solution, the wavelength undergoes a redshift because the refractive index in contact with the gold surface increases.

Table 3 reports a comparison of the difference in the resonance wavelength shifts due to the MIP deposition step for doped and not-treated platforms: the shift of the SPR peak after the functionalization process is slightly higher (about 25%) on the doped sensor configuration, compared to the one obtained on the not-treated sensor, pointing out once more the optical sensitivity increase of the treated SPR sensor.

**Figure 10:**
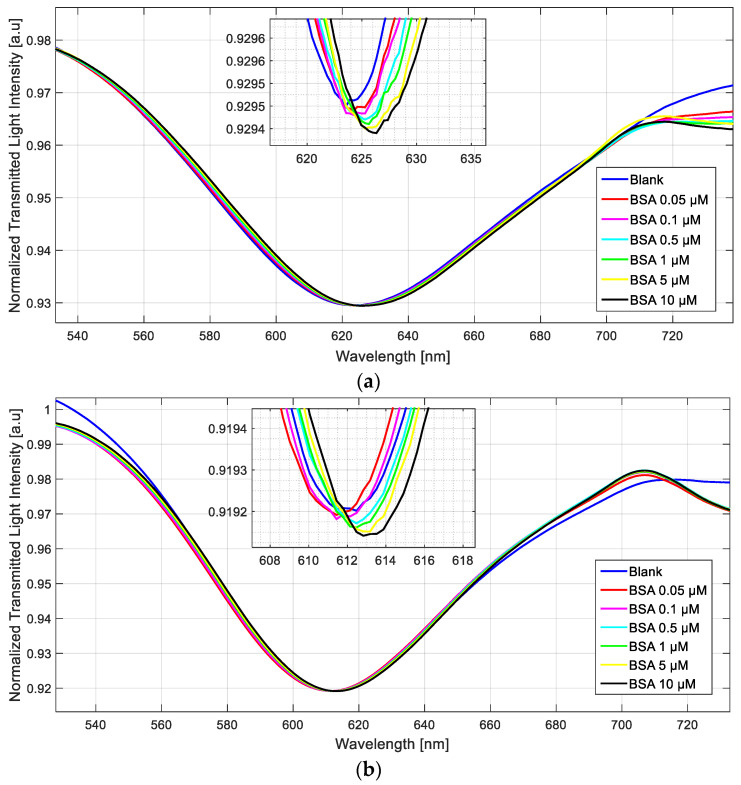
SPR spectra at different BSA concentrations in phosphate buffer, collected by a doped sensor (**a**) and by a not-treated sensor (**b**).

Figure 10 shows SPR spectra collected on a doped and a non-doped sensor with BSA solutions in the 0.05–10 µM concentration range.

Figure 10 shows a shift to higher resonance wavelength values (redshift) in both cases when the BSA concentration increases. More specifically, the doped sensor denotes a significant resonance wavelength shift even at very low concentrations (0.05 μM), while the not-treated sensor does not show any shift until 0.5 μM.

**Figure 11:**
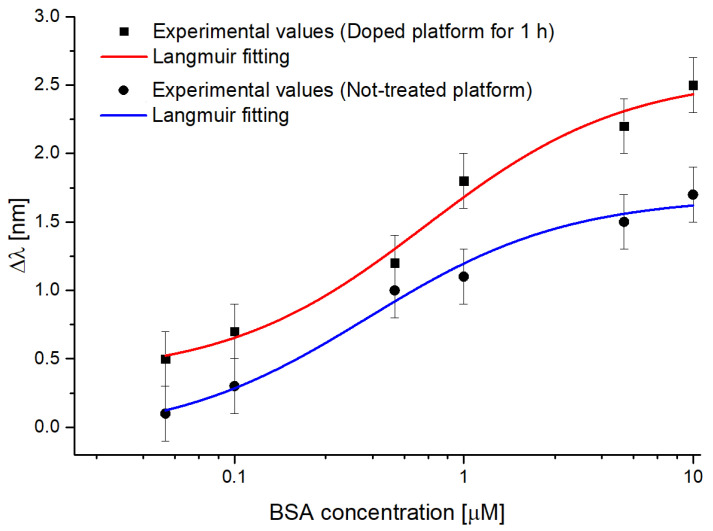
The resonance wavelength variations (calculated with respect to the blank) versus BSA concentration in phosphate buffer, with the error bars and Langmuir fitting in semi-log scale for doped and not-treated sensor.

Figure 11 shows the dose-response curves, relative to Figure 10, together with the Langmuir fitting to the experimental data, acquired with BSA solutions (0.05–10 μM) on a doped sensor (red curve) and a not-treated one (blue curve).

The experimental values were fitted by using the Langmuir model equation, which can be defined as follows:

(4) $${∆\lambda }_{c}=\lambda_{c}-\lambda_{0}={∆\lambda }_{max}\cdot \left(\frac{c}{K+c}\right)$$

where λ_c_ is the resonance wavelength at the BSA concentration *c*, $\lambda_{0}$ is the resonance wavelength at zero concentration (blank solution), ${\Delta \lambda }_{max}$ is the highest value of ∆λ_c_ that can be obtained at high concentration, and *K* is the dissociation constant.

Table 4 lists the Langmuir fitting parameters obtained using the Origin Pro program (Origin Lab. Corp., Northampton, MA, USA) relative to the fittings reported in Figure 11. To better fit the experimental values, the program automatically calculates the fitted curves’ parameters (listed in Table 4). These parameters are important for determining the chemical properties of the sensor, i.e., sensitivity at low concentrations, the limit of detection (LOD), and the affinity constant (K_aff_), which are reported in Table 5.

As it is clear from Table 5, the doping process led to a noticeable improvement in the binding performance in terms of the limit of detection (LOD). In fact, the LOD value evaluated in the case of the doped sensor halved with respect to the one obtained by the not-treated sensor and was calculated according to the following equation [41,56]:

(5) $$\mathrm{LOD}=\frac{3\cdot \sigma_{\Delta \lambda_{0}}}{\left(\Delta \lambda_{max}/K\right)}$$

where $\sigma_{\Delta \lambda_{0}}$ is the standard deviation of the blank (i.e., the standard error of $\Delta \lambda_{0}$) and $\Delta \lambda_{max}/K$ is the sensitivity at low concentrations. Moreover, in Figure 11, an increase in the overall resonance wavelength shift can be noticed, thus confirming once again that the doped sensor denotes better optical sensitivity than a not-treated one.

Finally, Table 6 summarizes a comparative analysis between the better BSA sensor configuration presented here and other BSA optical sensor configurations (state-of-the-art).

As shown in Table 6 below, the doped sensor’s LOD is lower than many other plasmonic sensor configurations. At the same time, it is the same order of magnitude as a fluorescence sensor.

## 5. Conclusions

The doping process carried out on PMMA-based plasmonic sensors caused an enhancement of the evanescent field and subsequently the SPR phenomenon, as demonstrated by the results obtained in both the optical characterization and the binding tests. In fact, from the preliminary test, the bulk sensitivity increased in the considered refractive index range (from 1.33 to 1.39) when a treated sensor was compared to a not-treated one. Chemical and morphological characterizations confirmed the PMMA modification in the first nanometers of the material. Subsequently, both kinds of sensors were tested in a chemical sensing scenario by considering a specific MIP layer synthesized for BSA detection. Similarly, the achieved results denoted an improvement of the sensor performance in terms of the detection limit, whose value decreased by a factor of about two concerning the doped sensor. Our approach tackles polymer doping from another angle, by using an already completed optical platform. The developed technique consists only in immerging the PMMA fibers into a solution: by virtue of its simplicity, it could be implemented in an industrial process in a cheaper and more readily available way. Moreover, no layer of iron oxide is formed in the polymer, hence, there is no need for sputtering, spinning, or other similar procedures that require specialized and expensive instrumentation.

In the future, the proposed doped sensor will be coupled with both alternative sensor configurations and with different receptors to diversify the determinable analytes and to reach lower detectable concentrations. Plus, other metal oxides could be examined as possible doping agents in order to further improve the plasmonic performance, along with the effects related to their properties, such as the magnetic metal oxides. Another possible future investigation could be focused on optimizing the duration of the doping procedure by shortening the amount of time it takes.

## Figures and Tables

**Table 1:** Chemical composition determined by XPS for not-treated fiber (A), treated without visible aggregates (B), or treated with aggregates observable on the surface (C) at different take-off angles (0°, 45°, and 60°, corresponding to different sampling depths from 10 to 5 nm). The oxygen component is deconvoluted into two or three components: the binding energy (eV) and the relative percentage are reported.

Sample	O (%)	O Components (B.E. (eV)—%)	C (%)	Fe (%)
A_0°	22.3	**O1: -** O2: 532.1–11.9 O3: 533.5–10.4	77.7	-
A_45°	20.7	**O1: -** O2: 532.1–10.5 O3: 533.5–10.2	79.3	-
A_60°	20.6	**O1: -** O2: 532.2–11.0 O3: 533.6–9.6	79.4	-
B_0°	23.6	**O1: 530.5–0.2** O2: 532.1–13.2 O3: 533.5–10.2	75.1	1.3
B_45°	20.2	**O1: 530.4–0.3** O2: 532.1 -11.2 O3: 533.5–8.7	78.6	1.2
B_60°	17.9	**O1: 530.5–0.7** O2: 532.2–11.4 O3: 533.6–5.8	80.5	1.6
C_0°	28.8	**O1: 530.2–3.2** O2: 531.7–13.8 O3: 533.4–11.8	64.6	6.6
C_45°	27.0	**O1: 530.1–5.9** O2: 531.8–15.5 O3: 533.5–5.6	65.0	8.0
C_60°	37.4	**O1: 530.0–7.9** O2: 531.7–21.9 O3: 533.4–7.6	49.9	12.7

**Table 2:** Fitting Parameters related to Equation (2) reported in Figure 7.

Configuration	A	B	C	Adj. R-Square
Not-treated Platform	8697.8	−22,303.1	14,275.1	0.999
Doped Platform for 1 h	7783.2	−19,684.2	12,409.5	0.998
Doped Platform for 3 h	6766.7	−16,981.6	10,612.2	0.998
Photoresist-coated Platform	6904.3	−17,358.8	10,871.4	0.997

**Table 3:** Resonance wavelength variation (Δλ) for doped and not-treated sensors relative to the MIP functionalization step.

Sensor	Δλ [nm]
Doped sensor	12
Not-treated sensor	9

**Table 4:** The Langmuir fitting parameters relative to BSA detection by two different types of platforms, i.e., doped platform and not-treated platform.

Configuration	Δλ_0_ [nm]		Δλ_max_ [nm]		K [µM]		Statistics	
	Value	St. Error	Value	St. Error	Value	St. Error	Χ^2^	R^2^
Doped sensor	0.3719	0.0852	2.5924	0.1241	0.4416	0.1997	0.3663	0.9779
Not-treated sensor	−0.0816	0.1267	1.6847	0.0841	0.3828	0.1192	0.2206	0.9783

**Table 5:** Sensors’ chemical parameters relative to the BSA detection in buffer.

Configuration	Sensitivity at Low Concentrations (Δλ_max_/K) [nm/μM]	LOD (3 × Standard Deviation of Blank (St. Error of Δλ_0_)/Sensitivity at Low Concentration) [μM]	K_aff_ (1/K) [μM^−1^]
Doped sensor	5.87	0.04	2.26
Not-treated sensor	4.40	0.09	2.61

**Table 6:** Comparison between different BSA sensor configurations.

Configuration	LOD [μM]	BSA Detection Range [μM]	Reference
Doped SPR D-shaped POF	0.04	0.05–10	This work
SPR-immunoassay	0.3	0.00015–0.15	[57]
SPR in a PMMA slab waveguide	0.0085	0.0085–1	[58]
SPR-D-shaped POF	0.37	0.37–6.5	[41]
Fluorescence sensor	0.01	0.01–2	[59]
Aggregation-induced emission biosensor coupled with graphene-oxide	0.4	0.4–1.5	[60]
SPR-MoS2 optical fiber	0.00436	0.00436–0.75	[61]
LSPR based on bimetallic nanoparticles	0.15 × 10^−6^	0.15–15 × 10^−6^	[62]
SPR-Kretschmann configuration	0.3	0.3–120	[63]

## Data Availability

Raw data supporting the conclusions of this article will be made available by authors upon request with undue reserve.
